# Prevalence and Determinants of Overweight and Obesity in Children and Adolescents from Migrant and Seasonal Farmworker Families in the United States—A Systematic Review and Qualitative Assessment

**DOI:** 10.3390/nu9030188

**Published:** 2017-02-24

**Authors:** Yuen Mei Lim, SuJin Song, Won O. Song

**Affiliations:** Department of Food Science and Human Nutrition, Michigan State University, 469 Wilson Road, Trout FSHN Building, East Lansing, MI 48824, USA; limyuen@anr.msu.edu (Y.M.L.); sjsong@msu.edu (S.S.)

**Keywords:** migrant and seasonal farmworker, children, adolescents, overweight, obesity, prevalence, health determinants

## Abstract

Overweight and obesity (OW/OB) is a pressing health concern among migrant and seasonal farmworker (MSFW) families in the US. The limited number of previously reported research on MSFW families suggests that their unique sociodemographic characteristics and lifestyle predispose them to poor health outcomes including OW/OB. We aimed to synthesize and assess available data on the prevalence and modifiable health determinants of OW/OB in MSFW children and adolescents. Literature search, study selection, data extraction and synthesis, and qualitative assessment of selected studies were performed independently by two authors. Ten cross-sectional studies met the inclusion criteria: articles or dissertations investigating prevalence and association between health determinants and OW/OB in MSFW children and adolescents (<20 years) in the US. The prevalence of OW, OB, and OW/OB ranged from 10%–33%, 15%–37%, and 31%–73%, respectively. Children’s education, household food insecurity, parents’ weight status, parents’ distorted perception of their children’s weight status, and parents’ participation in the federal nutrition assistance program were significantly associated with the children’s and adolescents’ risk of OW/OB. Promotion of culturally relevant public health programs and implementation of a systematic health surveillance plan for MSFWs and their children should be emphasized to combat OW/OB among MSFW children and adolescents.

## 1. Introduction

### 1.1. Overweight and Obesity in Children and Adolescents

The high prevalence of overweight and obesity (OW/OB) among children of all age groups is a pressing health concern in the US. At the national level, the prevalence of infants and toddlers (<2 years) with ≥97.7th percentile weight-for-recumbent length was 11.4% in 1999–2000 [1] and 7.1% in 2011–2012 [2]. The prevalence in Hispanics/Latinos of the same age group was 1.2 times higher during the same time periods [1,2]. Children and adolescents (2–19 years) are considered OW with a body mass index (BMI) between ≥85th and <95th percentile and obese with a BMI ≥95th percentile [2]. The national prevalence of OW/OB among children and adolescents was 23.0% in 1988–1994 [3] and 31.8% in 2011–2012 [4]. The prevalence was at least twice higher in Hispanics/Latinos of this age group during similar time periods [2,5]. Further, comparably high prevalence of OW/OB was reported in low-income children [6,7].

Childhood OW/OB is an important public health issue. Children at various ages have 2–10 times higher risk of being OW or obese in adulthood compared with normal weight children [8,9]. About 30% of obese preschool children and 50% of obese school-age children became obese as adults [8]. The risk ratio of adolescent OW/OB status persisting into adulthood increases to 4–15 times compared to normal weight children [9]. This persistence of childhood OW/OB can adversely impact health and quality of life with the development of acute and chronic non-communicable comorbidities [8,9,10,11,12,13].

According to an expert panel convened by the National Heart, Lung and Blood Institute, “OB is a complex multifactorial disease that develops from an interaction of genotype and the environment [14]”. There are numerous health determinants of OW/OB. Consumption of sugar-sweetened beverages, caloric-dense and high-fat foods, physical inactivity, short sleep duration, inflammation, and psychological disorders have consistently been cited in literature as health determinants of OW/OB in children and adolescents in the US [8].

### 1.2. Migrant and Seasonal Farmworkers and Their Children

Migrant and seasonal farmworkers (MSFWs) are agricultural workers engaged in farm work [15]. A seasonal farmworker is defined as “a person who during the preceding 12 months worked at least an aggregate of 25 or more days or parts of days in which some work was performed in farm work, earned at least half of his/her earned income from farm work, and was not employed in farm work year round by the same employer [15]”. A migrant farmworker is defined as “a seasonal farmworker who had to travel to do the farm work so that he/she was unable to return to his/her permanent residence within the same date [15]”. The exact enumeration of MSFW families and children is unknown due to their highly transient lifestyle, legal status, and the lack of systematic health surveillance and data collection [16,17,18]. However, various agencies estimate there are at least 300,000 children who work as farmworkers in the US [19,20]. Although MSFWs contribute significantly to the economic success of agricultural and food industries [17,21], MSFWs and their families are known subjects of social marginalization.

### 1.3. Health Problems in Migrant and Seasonal Farmworker (MSFWs) and Their Children

Existing literature reveals that MSFW families and especially their children are vulnerable to many chronic non-communicable diseases [22,23,24,25,26,27,28]. High food insecurity, a highly transient lifestyle, poor labor compensation for long work hours, poor English proficiency [17,22,29,30,31,32,33,34,35], substandard or limited housing and workplace provisions, harsh weather conditions [23,24,25,26,27,36,37,38,39], limited health care access, and acculturation [16,23,24,25,26,27,28,29,30,31,32,33,34,35,36,37,38,39] are some unique challenges they experience as a result of their low socioeconomic status, ethnicity, and farm work occupation. These challenges are believed to predispose MSFWs and especially their children to poor health outcomes such as cardiovascular diseases [24,34,37], gastrointestinal disorders [24,25,34,35], diabetes [24,25,26,34,35,37,39], hypertension [24,25,26,34,35,37,39], depression [24,25,26,28,34,37], cancer [26,34], asthma [24,25,26,34,35], anemia [25,26,28,34,39], lead [24] and pesticide poisoning [25,26,27,36], and OW/OB [24,25,26,27,28,34,37].

Common health determinants cited in OW/OB research on Hispanic/Latino children include children’s dietary intake [40,41,42,43], children’s demographic and socioeconomic characteristics [41,42,43,44], children’s physical activity [40,42,43], parents’ marital status [42,43], parents’ demographic and socioeconomic characteristics [40,42,43,44], parents’ perception of their children’s weight status [42], breastfeeding practices [43,44], and parents’ weight status [40,41,42,43,44]. Because the majority of MSFWs identify as Hispanics/Latinos [24,27,33,34,35,36,37,39,40,45,46,47,48,49,50,51,52,53,54,55], it is speculated that these health determinants may have a similar impact on the risk of MSFW children and adolescents being OW or obese. However, previously reported research on prevalence and health determinants of OW/OB in MSFW children and adolescents is limited.

### 1.4. Needs and Objectives of Systematic Review

Important overarching goals of Healthy People (HP) 2020 are the achievement of health equity, elimination of health disparities, and improvement of the health of as well as the promotion of quality of life, healthy development, and healthy behaviors among minority and underserved children [56]. Research studies focusing on OW/OB in MSFW families and children in the US have been small scale [48,50,53,54,57] and scattered [28,48,50,51,52,53,54,55,57,58]. To our knowledge, there is currently no available systematic review on the prevalence and health determinants of OW/OB in MSFW families and children. By systematically compiling, summarizing, and qualitatively assessing information on this topic of interest, the prevalence of OW/OB and the determinants that significantly influence the risk of being OW/OB in this hard-to-reach and marginalized population can be identified. Researchers and policymakers would have readily available access to evidence on this topic of interest to promote the development of culturally relevant public health policies and health promotion interventions that are focused on successfully modifying various health determinants of OW/OB among MSFW children and adolescents. These culturally relevant policies and interventions are necessary to ultimately achieve the overarching goals of HP 2020. Therefore, the aim of this systematic review was to synthesize and qualitatively assess the available data on the prevalence and modifiable health determinants of OW/OB in children and adolescents from MSFW families in the US.

## 2. Methods

### 2.1. Literature Search Plan

Literature search was performed by the first author in accordance with standards for systematic reviews from the Institute of Medicine [59], Patient-Centered Outcomes Research Institute [60], and Preferred Reporting Items for Systematic Reviews and Meta-Analyses statement [61] and guidelines for qualitative assessment of evidence from the Academy of Nutrition and Dietetics’ (AND) Evidence Analysis Manual: Steps in the Academy Evidence Analysis Process [62]. Michigan State University Libraries’ Search Plus search engine was used to perform a comprehensive search for literature on children and adolescents from MSFW families [63]. The following keywords were used in the literature search: OW, OB, and MSFW children. Additional relevant articles were identified by hand search of all articles extracted from the search engine. Supplementary Material A illustrates the detailed literature search plan.

### 2.2. Literature Selection

**Figure 1:**
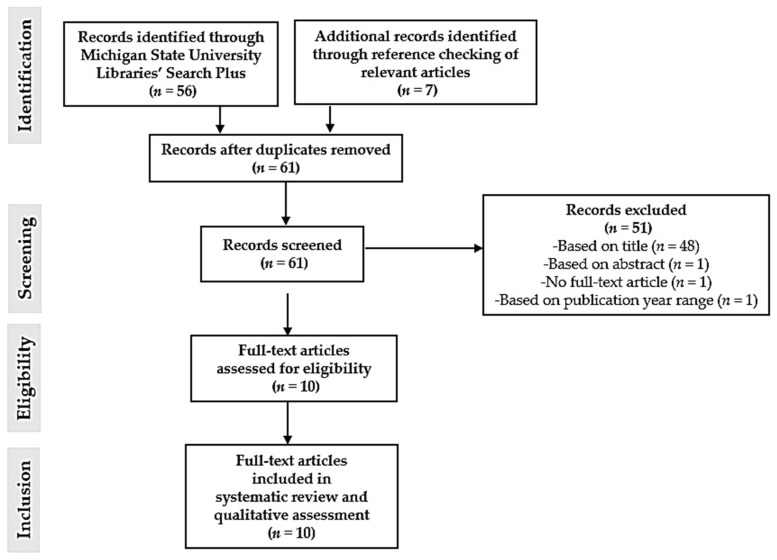
Literature search and selection process for studies included in this systematic review and qualitative assessment.

Literature selection was performed independently by the first two authors. Relevant articles were screened based on their titles and abstracts first, then based on pre-specified inclusion and exclusion criteria (Supplementary Material A). Subsequently, full-text versions of relevant articles were assessed for eligibility. Finally, the two authors mutually selected the articles for inclusion in the systematic review and qualitative assessment. Figure 1 shows a flow chart of the literature search and selection process.

Articles that were published either as a journal article or dissertation, in English language, published from any time frame up to August 2015, included children and adolescents of MSFWs (<20 years) of any health status, and addressed the outcome of interest which is OW/OB were included. OW/OB in MSFW were measured by the age- and sex-specific percentiles for BMI. Infants and toddlers (<2 years) were considered OW with a BMI ≥95th or ≥97.7th percentile according to the Centers for Disease Control and Prevention (CDC) 2000 [2] or World Health Organization (WHO) 2006 growth charts and standards [64], respectively. No recommended definitions of OB are available for infants and toddlers of this age range [2,65]. Children and adolescents (2–19 years) were considered OW with a BMI between ≥85th and <95th percentile [2,64] and obese with a BMI ≥95th percentile [2,65]. In addition, studies performed in any setting in the US, with study samples of any size, no restriction on attrition rates, and preferably quantitative over qualitative design were eligible for inclusion. Articles that were published either as a book/ebook, book chapter, book review, government document, newsletter, newspaper article, in any language other than English, published in any other country other than the US, published after August 2015, included adults (≥20 years) and children and adolescents (<20 years) that were not from MSFW families, and did not address the outcome of interest were excluded.

### 2.3. Data Extraction and Synthesis of Selected Studies

Extraction and synthesis of pertinent data from the selected studies occurred between September 2015 and February 2016. The first author conducted data extraction using a structured data collection table. The following data were extracted from each article: study characteristics including first author’s last name, publication year, study location, study year, study design; study participant characteristics including sample size, sample type (e.g., MSFW children), age, gender, parents’ ethnicity ( e.g., Hispanic/Latino), parents’ farmworker designation, citizenship (e.g., born in the US or Mexican-descent), and enrollment in Migrant Head Start (MHS); outcome of interest (e.g., prevalence of OW/OB) and its definitions (e.g., definitions of OW/OB according to CDC 2000 and WHO 2006 growth charts and standards), independent variables (e.g., types of health determinants) and its association with OW/OB, statistical analysis (e.g., statistical methods used to measure association) and its findings (e.g., odds ratio, correlation coefficient, *p* values), and conclusion of the study (e.g., whether health determinants and OW/OB are associated or not, the significance of the association—statistically significant or non-significant, and whether the independent variable being studied increases or decreases the risk of OW/OB—positive if an independent variable increases the risk of OW/OB and negative if an independent variable decreases the risk of OW/OB).

### 2.4. Qualitative Assessment of Selected Studies

Qualitative assessment of the selected studies was performed by the first two authors independently following the AND’s Quality Criteria Checklist for Primary Research from the Evidence Analysis Manual: Steps in the Academy Evidence Analysis Process [62]. Disagreements were discussed and resolved between the first two authors. The contents of the Quality Criteria Checklist for Primary Research and the methods for assessing quality and risk of bias as well as for assigning a recommended grade for the evidence of each study are described in the section titled “Grade the Strength of the Evidence Supporting the Conclusion Statement” in the AND’s Evidence Analysis Manual: Steps in the Academy Evidence Analysis Process [62].

## 3. Results

### 3.1. Literature Search Results and Characteristics of Study Population

Initial literature search identified 63 relevant articles to be screened. After removal of duplicates and screening based on title, abstract contents, and pre-specified inclusion and exclusion criteria, ten full-text articles were assessed for eligibility to be included in the systematic review and for qualitative assessment [28,48,50,51,52,53,54,55,57,58]. Assessment of the full-text articles resulted in all ten articles remaining eligible for systematic review and qualitative assessment (Figure 1). Supplementary Material A shows the contents of the complete search plan and results of primary research articles for our study using the template provided by the AND’s Evidence Analysis Manual: Steps in the Academy Evidence Analysis Process.

The characteristics of the study populations included in the ten assessed articles are shown in Table 1. The total number of children and adolescents was 3157 (range *n* = 20–1347) [28,48,50,51,52,53,54,55,57,58]. The age of children and adolescents ranged from 0 to 18 years [28,48,50,51,52,53,54,55,57,58]. The ratio of male to female children and adolescents in each study was balanced overall. A majority of the children and adolescents had parents whose ethnicity was either Hispanic or Latino [48,50,51,52,53,54,55,57]. Of the total children and adolescents, 75% had parents whose farm work was migrant and seasonal [51,52,54,57,58] and the remainder were migrant in nature [28,48,50,53,55]. A total of 46% of the children and adolescents were MHS enrollees [54,57,58].

### 3.2. Prevalence of OW/OB

The prevalence rates of OW/OB in children and adolescents (0–18 years) from MSFW families are shown in Table 1 [28,48,50,51,52,53,54,55,57,58]. Overall, the prevalence rates of OW for children and adolescents (2–18 years) range from as low as 10% to as high as 33% [28,48,51,52,53,54,58], OB 15% to 37% [28,48,51,54,58], and OW/OB 31% to 73% [28,48,50,51,54,57,58]. These prevalence rates do not include rates categorized according to age groups [50,53], gender [28], or school enrollment year [58]. Only one study reported the prevalence rate of OW for infants and toddlers (<2 years) being 7% [53].

### 3.3. Health Determinants of OW/OB

Table 2 shows the eight modifiable health determinants and characteristics reported in the studies assessing the association between the modifiable health determinants and OW/OB in children and adolescents from MSFW families in the US. Kilanowski [50] investigated the association between children’s dietary intake and their risk of OW/OB. Daily intake of five food groups from the United States Department of Agriculture’s (USDA) Food Guide Pyramid was not significantly associated with the risk of OW/OB in migrant children and adolescents. The number of OW and obese children and adolescents who met all five food group recommendations was twice that of those who were underweight or normal weight.

Two studies reported statistically significant associations between children’s education and their risk of OW/OB [55,58]. Lee and Song [58] used enrollment duration in MHS as a measure of education and found that children who had longer enrollment were significantly less OW than those whose enrollment was shorter. Rosado et al. [55] used school grade level as a measure of education and found children and adolescents in higher school grade levels were less likely to be OW or obese than those in lower school grade levels.

Three studies examined the association between household food insecurity and children’s risk of OW/OB and reported mixed findings [50,54,57]. Borre et al. [57] found OW/OB to be significantly less prevalent among MHS children in food-insecure households than those in food-secure households. On the other hand, Kilanowski [50] found OW/OB to be more prevalent among migrant children and adolescents in high food-insecure households. Meanwhile, Song et al. [54] found no significant association between the different levels of household food insecurity and the children’s weight status.

Song et al. [54] also investigated the associations of parents’ education, parents’ perception of their children’s weight status, and parents’ health insurance with their children’s risk of OW/OB. Using parents’ nutrition knowledge as a measure of their education, no significant association was found between the number of nutrition knowledge questions parents of MHS children could answer correctly and their children’s weight status. The lack of health insurance was also not significantly associated with the risk of OW/OB. The lack of health insurance was more prevalent in families with children who were OW or obese compared to families with children who were non-obese. Parents’ distorted perception of their children’s weight status, however, was significantly associated with their children’s likelihood of being OW/OB. Parents of OW or obese children were more likely to misperceive their children’s weight status compared with parents whose children were non-obese.

Two studies examined the association between parents’ weight status and their children’s risk of OW/OB [54,55]. Both studies used parents’ BMI as a measure of their weight status and reported statistically significant associations of this health determinant with their children’s OW/OB status. Parents’ BMI significantly predicted migrant children’s and adolescents’ BMI percentile and OW/OB status in Rosado et al.’s study [55]. Similarly, in Song et al.’s study [54], parents of MHS children who were OW or obese were significantly more likely to have OW or obese children than non-obese children.

Lee and Song [58] investigated the association between parents’ participation in the federal nutrition assistance program and their children’s risk of OW/OB using Supplemental Nutrition Assistance Program (SNAP) participation as a measure of the federal nutrition assistance program participation. The children of MSFW families that received SNAP benefits were found to be significantly less likely to be OW or obese compared to those from families that did not participate in SNAP.

### 3.4. Qualitative Assessment of Studies Included in Systematic Review

Table 3 shows the detailed results of the qualitative assessment of studies included in the systematic review. All the included studies were cross-sectional in design [28,48,50,51,52,53,54,55,57,58]. According to the Grade the Strength of the Evidence Supporting the Conclusion Statement Guidelines from the Evidence Analysis Manual, grade II is recommended for qualitative assessment of the studies included for systematic review. Six studies were rated “+” for their strength of evidence [28,48,51,53,55,58]. The remaining four were of “Ø” rating [50,52,54,57], and none of the studies were given a “–“ rating.

## 4. Discussion

This systematic review assessed the prevalence of OW/OB and its significant associations with health determinants in MSFW children and adolescents in the US using data from ten cross-sectional studies. The prevalence of OW/OB for children and adolescents (2–18 years) of MSFWs was between 31% and 73% [28,48,50,51,54,57,58] and 7% for infants and toddlers ages (<2 years) [53]. Among the eight modifiable health determinants, children’s education [55,58], household food insecurity [57], parents’ distorted perception of their children’s weight status [54], parents’ weight status [54,55], and parents’ participation in the federal nutrition assistance program were significantly associated with the children’s and adolescents’ risk of OW/OB [58].

### 4.1. Prevalence of OW/OB

The prevalence of OW/OB among MSFW children and adolescents identified in this systematic review was consistently higher than the national prevalence [3,4] and those in low-income households [6,7], but comparable to those of Hispanic/Latino ethnicity [42,66]. The prevalence of OW/OB among MSFW children and adolescents in our systematic review was 31%–73%, which was significantly higher than those previously reported nationally (23% and 32%) [3,4] and in children and adolescents who have similar income status (27% and 32%) [6,7]. Our finding was also comparable with previous studies of Hispanic/Latino children and adolescents that reported prevalence rates of OW/OB between 43% and 61% [2,5,42,66]. This could be a result of the fact that a majority of MSFW children and adolescents are Hispanics/Latinos. The range of prevalence rates from our systematic review, however, may have resulted from differences in the sample size, the chosen age group, and the geographic regions where each study was conducted. Along with the lack of sample size disclosure [49,51,54] and standardized BMI cut-off points used in each study to define OW/OB [28,48,50,51,52,53,54,55,57,58], these factors altogether can contribute to an under- or over-estimation of its prevalence.

Our finding that 7% of infants and toddlers had a BMI ≥97.7th percentile weight-for-recumbent length was surprisingly lower than the prevalence reported for infants and toddlers of Hispanic/Latino ethnicity [1,2,7] but similar to the prevalence reported for the general population of the same age group [1,2]. Previous studies of Hispanic/Latino infants and toddlers reported prevalence rates of BMI ≥97.7th percentile weight-for-recumbent length as high as 11 times higher than our systematic review finding [1,2,7]. This difference could be attributed to the different sample size used for prevalence calculation. However, this finding cannot be generalized because only one study assessed the prevalence of BMI ≥97.7th percentile weight-for-recumbent length in a very small sample of MSFW infants and toddlers [53]. Due to the limited sample size of the study, sex- or age-stratified information on the prevalence of OW/OB could not be founded. Further research investigating the prevalence of OW/OB in younger MSFW children is needed to more accurately assess the severity of OW/OB in this age group because the risk of childhood OW/OB development is significantly increased in the first 1000 days of life [67].

### 4.2. Health Determinants of OW/OB

Children’s education was inversely associated with their risk of OW/OB. This finding can be explained by the incorporation of a nutrition and physical activity component in a school curriculum. The decreased OW/OB risk could be attributed to MSFW children’s and adolescents’ compliance with nutrition and physical activity programs through MHS enrollment [58]. MHS is a childcare and federal preschool program for children of eligible farmworkers [68]. The program is designed to promote school readiness [68]. Also, it is speculated that as MSFW children and adolescents progress through school, the increased amount and variety of physical activity they receive may reduce their risk of OW/OB. However, other studies of Hispanic/Latino children have found no association with OW/OB using other measures of physical activity such as television viewing and time spent playing outdoors [40,42,43]. Future studies should consider including physical activity as an independent variable to assess its association with OW/OB.

An interesting finding was household food insecurity’s inverse association with children’s and adolescents’ risk of OW/OB [57]. This finding was inconsistent with other studies that demonstrated higher prevalence of OW/OB in highly food-insecure households [43,50] and no significant association between food security and OW/OB [54]. The mixed findings of these studies could be attributed to the use of different household food insecurity assessment tools and different sample sizes.

Parents’ participation in the federal nutrition assistance program was also inversely associated with their children’s risk of OW/OB. Using SNAP participation as a measure of the federal nutrition assistance program participation, children from MSFW families that received SNAP benefits were significantly less likely to become OW or obese compared to those in families that did not receive benefits [58]. Our finding was consistent with those published in a USDA report, where the increase in BMI or the risk of OW/OB in low-income children was linked to the usage of SNAP benefits [69]. It is possible that weight status changes can be prevented through increased food access with longer participation and use of SNAP benefits [69]. Adult women participating in the SNAP program, however, were more likely to be obese than those not participating in SNAP [69]. Therefore, it is important to specify the recipient of SNAP benefits in future studies because gender and age can have differential impacts on the outcomes of SNAP benefits usage [70] and the majority of MSFW mothers are responsible for meal preparation and food purchases in the household [46].

Parents’ distorted perception of their children’s weight status and parents’ weight status were positively associated with their children’s risk of OW/OB. Our findings were consistent with those of several previous studies, where maternal BMI was a significant predictor of OW/OB [40,41,42,43] among Hispanic/Latino children. Further, OW or obese parents of Hispanic/Latino ethnicity were more likely to misclassify their OW or obese children as having normal weight [42,71]. These findings may be explained by the cultural perception that being OW is a symbol of greater health [72,73]. Along with their low socioeconomic status and identification as Hispanics/Latinos, MSFW parents’ cultural, dietary, and social beliefs and practices may prevent efforts in managing their children’s OW/OB status. Future studies should explore the role of all family members because the studies included in this systematic review did not specify the gender of MSFW parents when assessing their children’s risk of OW/OB.

Children’s dietary intake, parents’ education, and parents’ health insurance were not significantly associated with the risk of OW/OB in this specific population. The finding on children’s dietary intake was inconsistent with previous studies in Mexican-American [40,43,44] and Hispanic/Latino [42] children and adolescents. Using diet quality as a measure of children’s dietary intake, Mexican-American children and adolescents who selected unhealthy choices of foods and beverages to consume regularly were significantly more likely to have an OW status [40] or be OW/OB [43] compared with those whose consumption was infrequent. The native diet of young Hispanic/Latino children, on the other hand, was not significantly associated with the children’s risk of OW [42]. These mixed findings could be attributed to the use of different measures of dietary intake and the different ages and nativity statuses of the population. The finding on parents’ education was consistent with those of previous studies, where mother’s education level was not statistically significantly associated with the risk of OW/OB in either Mexican-American [40,43,44] or Hispanic/Latino [42] children and adolescents. The finding on parents’ health insurance is inconsistent with previous research on Mexican-born children experiencing higher socioeconomic status [43]. It is speculated that the contrast between this finding with those of previous studies could be attributed to the differences in the studied population’s socioeconomic status and food insecurity.

### 4.3. Strengths and Limitations

Several limitations exist in this review paper. First, the findings do not include certain unmodifiable sociodemographic, biological, and psychological health determinants of OW/OB. To achieve the overarching goals of HP 2020, interventions in the form of culturally relevant policies, programs, and information dissemination are needed to ensure the successful modification of health determinants that significantly contribute to the risk of childhood OW/OB [56]. Second, identification of any causal relationship between the determinants and the health outcome was not possible due to the cross-sectional design of all studies included in this systematic review [28,48,50,51,52,53,54,55,57,58]. In addition, determination of OW/OB trends cannot be made with the use of data from cross-sectional studies. Longitudinal data in this population could contribute to the determination of trends and identification of causal relationships. However, it is difficult to collect longitudinal data especially in this population due to their highly mobile lifestyle [16,17]. Third, the findings of this systematic review may not be representative of the entire MSFW children and adolescent population residing in the US. Available literature on OW/OB in MSFW children and adolescents has been geographically concentrated in a few states in the US [28,48,50,51,52,53,54,55,57,58] and based on small sample sizes [48,50,53,54,57]. Fourth, only published data from primary sources were included in this systematic review. The availability of published secondary sources featuring data specifically on this topic of interest was limited. Furthermore, it was not possible to perform qualitative assessment of secondary sources using the guidelines from the AND Evidence Analysis Manual: Steps in the Academy Evidence Analysis Process [62]. The quality criteria checklist from the AND Evidence Analysis Manual to qualitatively assess for scientific validity are available only for primary research or review articles. Fifth, the response rates and sampling strategies of the included studies were not reported due to the lack of sampling frame, small sample size, and their study design. Therefore, the generalizability of the findings from the included studies is limited. The lack of sampling frame is one of the unique challenges of using a hard-to-reach population and response rates are typically low or not reported when convenience sampling is utilized for data collection. Future studies emphasizing longitudinal data collection, larger sample sizes and recruitment throughout the nation are needed to determine causal relationships and trends of OW/OB prevalence in MSFW children and adolescents in the US.

Despite these limitations, this systematic review has several strengths. This is the first systematic review that synthesized and qualitatively assessed available data on the prevalence and health determinants of OW/OB in MSFW children and adolescents in the US. The systematic review identified modifiable health determinants that significantly contribute to the risk of OW/OB among MSFW children and adolescents [54,55,57,58]. Identification of modifiable health determinants is an important step in ameliorating OW/OB in children and adolescents and ultimately achieving HP 2020 overarching goals. While publication bias is likely due to the limited previous research on OW/OB in MSFW families, publication bias is, however, limited as the included studies show evidence of publication of both significant and non-significant findings on this topic of interest. We also considered the comparison of our findings with those from studies involving Mexican-American and Hispanic/Latino children and adolescents a strength of this paper. By comparing our findings with those of studies using other populations with similar socioeconomic characteristics and ethnicity, the prevalence and significant health determinants that uniquely influence the risk of OW/OB in MSFW children and adolescents were identified. Studies investigating the health status of MSFW families and their children have shown that the majority of MSFW children and adolescents have low socioeconomic status and are of Hispanic/Latino ethnicity [24,27,33,34,35,36,37,39,40,45,46,47,48,49,50,51,52,53,54,55].

## 5. Conclusions

Research in this discipline remains limited and overweight and obesity (OW/OB) is still a prevalent and concerning health problem among children and adolescents of migrant and seasonal farmworkers (MSFWs). Findings from this systematic review indicate disproportionately high rates of OW/OB among MSFW children and adolescents. Modifiable health determinants significantly associated with OW/OB were children’s education, household food insecurity, parents’ distorted perception of their children’s weight status, parents’ weight status and parents’ participation in the federal nutrition assistance program. This systematic review provides researchers and policymakers with evidence to promote culturally relevant interventions in terms of health programs, public policies, and information dissemination focused on modifying health determinants that significantly influence the risk of OW/OB in MSFW children and adolescents. Alongside these needed interventions, the establishment of a systematic health surveillance plan in this population would provide the relevant data that would likely play a significant contributory role in ameliorating childhood OW/OB and achieving the overarching goals of Healthy People 2020.

## Figures and Tables

**Table 1:** Overweight and obesity prevalence.

First Author, Publication Year, Location	Study Year, Study Design	*n*	Characteristics of MSFW Children and Adolescents	Definitions of OW/OB *	Prevalence of OW/OB
**Borre, 2010, North Carolina [57]**	2005, Cross-sectional	Food secure (*n* = 13), Food insecure (*n* = 17)	Migrant and seasonal, East Coast MHS enrollees, Latino parents, 2–7 years, M: 43%, F: 57%	OW: BMI ≥85th and <95th percentile; OB: BMI ≥95th percentile	OW/OB in food-insecure and -secure children are 33% and 73%, respectively.
**Grzywacz, 2014, North Carolina [51]**	2011-2012, Cross-sectional	242 mother-child dyads	Migrant and seasonal, low-income, Latino parents, 99% born in US, 2.5–3.5 years, M: 48%, F: 52%	OW: BMI ≥85th and <95th percentile; OB: BMI ≥95th percentile	OW: 19.8%. OB: 21.9%. OW/OB: 41.7%.
**Kilanowski, 2006, Ohio [48]**	N/A, Cross-sectional	20	Migrant, Hispanic/Latino parents, 4 months–12 years, M: 50%, F: 50%	OW: BMI ≥85th and <95th percentile; OB: BMI ≥95th percentile	OW: 19%. OB: 33%. OW/OB: 52%.
**Kilanowski, 2007, Ohio [53]**	N/A, Cross-sectional	52	Migrant, 94% Hispanic parents, 4 months–12 years, M: 52%, F: 48%	OW: BMI ≥85th and <95th percentile; OB: BMI ≥95th percentile	Overall OW: 33%. OW 6–11 years (*n* = none): 41%. OW infants and toddlers (*n* = 16): 7%.
**Kilanowski, 2012, Ohio and Michigan [50]**	N/A, Cross-sectional	58 parent-child dyads	Migrant, Hispanic, 2–13 years, M: N/A, F: N/A	OW: BMI ≥85th percentile; OB: BMI ≥95th percentile	OW/OB 2–5 years: 50%. OW/OB 6–11 years: 45%.
**Lee, 2015, Michigan [58]**	2012-2013, Cross-sectional	1357	Migrant and seasonal, MHS enrollees, multirace parents, 0–6 years, M: 48%, F: 52%	OW: BMI ≥85th and <95th percentile; OB: BMI ≥95th percentile	Overall OW (*n* = 167 out of 1357): 16.1%. OW at 1 year enrollment (*n* = 92 out of 638): 19.1%, at 2 years enrollment: 16.3% (*n* = 39 out of 293). At >3 years enrollment (*n* = 36 out of 426): 11.3%. Overall OB (*n* = 157 out of 1357): 15.1%. OB at 1 year enrollment (*n* = 62 out of 638): 12.9%, at 2 years enrollment (*n* = 45 out of 293): 18.8%. At >3 years enrollment (*n* = 50 out of 426): 15.7%. OW/OB: 31.2%.
**Markowitz, 2005, New Jersey [52]**	1997-2004, Cross-sectional	667	Migrant and seasonal, Hispanic Mexican parents, 2–18 years, M: 54%, F: 46%	OW: BMI ≥95th percentile	OW: 20.1%.
**Nichols, 2014, Georgia [28]**	2011, Cross-sectional	183	Migrant, parents’ ethnicity not mentioned, 0–16 years, M: 50%, F: 50%	OW: BMI ≥85th and <95th percentile; OB: BMI ≥95th percentile	OW: 17.6%. OB: 37.4%. OW/OB: 55%. Males (45.6%) had higher OB prevalence than females (29.4%); 2–5 years (21.7%) had lower OB prevalence than 6–11 years (47.8%) and 12–16 years (43.5%).
**Rosado, 2013, Florida [55]**	2010–2011, Cross-sectional	472	Migrant, Latino parents, US-born of Mexican descent, 3–16 years, M: 51%, F: 49%	OW: BMI ≥85th and <95th percentile; OB: BMI ≥95th percentile	OW: 20.1%. OB: 27%. OW/OB: 47.1%.
**Song, 2015, Michigan [54]**	2013, Cross-sectional	76 families with ≥1 child	Migrant and seasonal, MHS enrollees, Hispanic/Latino parents, 0–5 years, M: 46%, F: 54%	OW: BMI ≥85th and <95th percentile; OB: BMI ≥95th percentile	OW: 10%. OB: 31%. OW/OB: 41%.

BMI, body mass index; F, female; M, male; MHS, Migrant Head Start; MSFW, migrant and seasonal farmworker; *n*, sample size; N/A, not applicable; OW, overweight; OB, obesity; OW/OB, overweight and obesity; US, United States. * OW/OB definitions were based on the Centers for Disease Control and Prevention 2000 Growth Charts and its corresponding age- and sex-specific percentiles for BMI.

**Table 2:** Health determinants of overweight and obesity.

Health Determinant	First Author, Publication Year, Location	Study Year, Study Design	*n*	Characteristics of MSFW Children and Adolescents	OW/OB Definitions *	Independent Variable	Independent Variable’s Association with OW/OB	Conclusion
**Dietary Intake**	Kilanowski, 2012, Ohio and Michigan [50]	N/A, Cross-sectional	58 parent-child dyads	Migrant, Hispanic, 2–13 years, M: N/A, F: N/A	OW: BMI ≥85th percentile; OB: BMI ≥95th percentile	Daily intake of five food groups on USDA Food Guide Pyramid	Children who met all five food group recommendations: 13% (*n* = 16) were UW or NW, 34% (*n* = 11) were OW, 33% (*n* = 12) were obese, *p* > 0.05.	No significant association
**Education**	Lee, 2015, Michigan [58]	2012-2013, Cross-sectional	1357	Migrant and seasonal, MHS enrollees, multirace parents, 0–6 years, M: 48%, F: 52%	OW: BMI ≥85th and <95th percentile; OB: BMI ≥95th percentile	Longer enrollment in MHS	Children who attended MHS for ≥3 years were significantly less OW than those who attended for 1 year (b coefficient = −0.70, OR = 0.50, *p* < 0.05).	Associated, significant (−)
	Rosado, 2013, Florida [55]	2010-2011, Cross-sectional	472	Migrant, Latino parents, US-born of Mexican descent, 3–16 years, M: 51%, F: 49%	OW: BMI ≥85th and <95th percentile; OB: BMI ≥95th percentile	Higher school grade level	Compared to preschool aged children, those in elementary school (multiple regression coefficient = 0.886, *p* = 0.030) are significantly more likely to be OW or obese than NW.	Associated, significant (−)
**Household Food Insecurity**	Borre, 2010, North Carolina [57]	2005, Cross-sectional	Food secure (*n* = 13) Food insecure (*n* = 17)	Migrant and seasonal, East Coast MHS enrollees, Latino parents, 2–7 years, M: 43%, F: 57%	OW: BMI ≥85th and <95th percentile; OB: BMI ≥95th percentile	Food-insecure households (Assessed by USDA 18-Item Household Food Security Module)	Of 57% food-insecure children, 33% were OW or obese, *p* < 0.01. Of 43% food-secure children, 73% were OW or obese, *p <* 0.01.	Associated, significant (−)
	Kilanowski, 2012, Ohio and Michigan [50]	N/A, Cross-sectional	58 parent-child dyads	Migrant, Hispanic, 2–13 years, M: N/A, F: N/A	OW: BMI ≥85th percentile; OB: BMI ≥95th percentile	Food-insecure households (Assessed by 5-Item Short Form US Household Food Security Scale)	Low or very low food security experienced by 75% (*n* = 12) of OW children and 53% (*n* = 15) of obese children as opposed to 48% (*n* = 21) of UW or NW children.	Associated, statistical significance not reported (+)
	Song, 2015, Michigan [54]	2013, Cross-sectional	76 families with ≥1 child	Migrant and seasonal, MHS enrollees, Hispanic/Latino parents, 0–5 years, M: 46%, F: 54%	OW: BMI ≥85th and <95th percentile; OB: BMI ≥95th percentile	Food-insecure households (Assessed by US Household Food Security Survey Module)	Different levels of food security status of household were not significantly associated with children’s weight status, *p =* 0.286.	No significant association
**Parents’ Education**	Song, 2015, Michigan [54]	2013, Cross-sectional	76 families with ≥1 child	Migrant and seasonal, MHS enrollees, Hispanic/Latino parents, 0–5 years, M: 46%, F: 54%	OW: BMI ≥85th and <95th percentile; OB: BMI ≥95th percentile	Parents’ nutrition knowledge	Number of correct answers (0, 1, 2, 3, 4, and 5) parents obtained from nutrition knowledge questions were not significantly associated with children’s weight status, *p* = 0.477; ≤50% parents answered most nutrition knowledge questions correctly.	No significant association
**Parents’ Health Insurance**	Song, 2015, Michigan [54]	2013, Cross-sectional	76 families with ≥1 child	Migrant and seasonal, MHS enrollees, Hispanic/Latino parents, 0–5 years, M: 46%, F: 54%	OW: BMI ≥85th and <95th percentile; OB: BMI ≥95th percentile	No health insurance	83% families with OW/OB children had no health insurance compared to 61% non-obese children, *p* = 0.066.	No significant association
**Parents’ Perception of Their Children’s Weight Status**	Song, 2015, Michigan [54]	2013, Cross-sectional	76 families with ≥1 child	Migrant and seasonal, MHS enrollees, Hispanic/Latino parents, 0–5 years, M: 46%, F: 54%	OW: BMI ≥85th and <95th percentile; OB: BMI ≥95th percentile	Parents’ distorted perception of their children’s weight status	Prevalence of parents who perceived their children to be OW, obese, UW, and NW are 6%, 0%, 3%, and 91%, respectively. Actual prevalence of OW, OB, UW, and NW children are 10%, 31%, 6%, and 53%, respectively. More parents of OW/OB children (100%) were misperceiving their children’s weight status than parents of non-obese children (15%), *p* < 0.001.	Associated, significant (+)
**Parents’ Weight Status**	Rosado, 2013, Florida [55]	2010–2011, Cross-sectional	472	Migrant, Latino parents, US-born of Mexican descent, 3–16 years, M: 51%, F: 49%	OW: BMI ≥85th and <95th percentile; OB: BMI ≥95th percentile	Parent BMI	Parents’ BMI is a significant predictor of children’s BMI percentile (multiple regression coefficient = 0.612, *p* = 0.030), OW/OB (multiple regression coefficient = 0.061, *p =* 0.007), and OB (multiple regression coefficient = 0.101, *p <* 0.001) status.	Associated, significant (+)
	Song, 2015, Michigan [54]	2013, Cross-sectional	76 families with ≥1 child	Migrant and seasonal, MHS enrollees, Hispanic/Latino parents, 0–5 years, M: 46%, F: 54%	OW: BMI ≥85th and <95th percentile; OB: BMI ≥95th percentile	Parent BMI	97% OW/OB children had parents who are also OW/OB compared to 64% non-obese children, *p* = 0.003.	Associated, significant (+)
**Parents’ Participation in Federal Nutrition Assistance Program**	Lee, 2015, Michigan [58]	2012–2013, Cross-sectional	1357	Migrant and seasonal, MHS enrollees, multirace parents, 0–6 years, M: 48%, F: 52%	OW: BMI ≥85th and <95th percentile; OB: BMI ≥95th percentile	SNAP participation	MHS children whose family received SNAP benefits were significantly less likely to be OW or obese (b coefficient = −0.41, OR = 0.67, *p* < 0.05) compared to those whose families did not receive SNAP benefits.	Associated, significant (−)

BMI, body mass index; F, female; M, male; MHS, Migrant Head Start; MSFW, migrant and seasonal farmworker; *n*, sample size; N/A, not applicable; OW, overweight; OB, obesity; OW/OB, overweight and obesity; OR, odds ratio; SNAP, Supplemental Nutrition Assistance Program; UW, underweight; NW, normal weight; US, United States; USDA, United States Department of Agriculture; +, independent variable increases the risk of OW/OB; −, independent variable decreases the risk of OW/OB. * OW/OB definitions were based on the Centers for Disease Control and Prevention 2000 Growth Charts and its corresponding age- and sex-specific percentiles for BMI.

**Table 3:** Qualitative assessment of studies included in the systematic review ^1^.

First Author, Publication Year	Design	Q1	Q2	Q3	Q4	Q5	Q6	Q7	Q8	Q9	Q10	QA
Borre, 2010 [57]	Cross-sectional	Y	Y	N	Y	UC	Y	Y	Y	Y	Y	Ø
Grzywacz, 2014 [51]	Cross-sectional	Y	Y	N/A	N	UC	Y	Y	Y	Y	Y	+
Kilanowski, 2006 [48]	Cross-sectional	Y	Y	N/A	N	UC	Y	Y	Y	Y	Y	+
Kilanowski, 2007 [53]	Cross-sectional	Y	Y	N/A	Y	UC	Y	Y	Y	Y	Y	+
Kilanowski, 2012 [50]	Cross-sectional	Y	Y	N	Y	UC	Y	Y	Y	Y	Y	Ø
Lee, 2015 [58]	Cross-sectional	Y	Y	Y	N	UC	Y	Y	Y	Y	Y	+
Markowitz, 2005 [52]	Cross-sectional	Y	Y	N	Y	UC	Y	Y	Y	Y	Y	Ø
Nichols, 2014 [28]	Cross-sectional	Y	Y	N/A	N	UC	Y	Y	Y	Y	Y	+
Rosado, 2013 [55]	Cross-sectional	Y	Y	Y	N	UC	Y	Y	Y	Y	Y	+
Song, 2015 [54]	Cross-sectional	Y	Y	N	Y	UC	Y	Y	Y	Y	Y	Ø

^1^ For the qualitative assessment of articles, the Academy of Nutrition and Dietetics Quality Criteria Checklist for Primary Research from the Evidence Analysis Manual was used; Y = Yes; N = No; UC = Unclear; N/A = Not applicable; Q1 = Was the research question clearly stated? Q2 = Was the selection of study subjects/patients free from bias? Q3 = Were study groups comparable? Q4 = Was method of handling withdrawals described? Q5 = Was blinding used to prevent introduction of bias? Q6 = Were intervention/therapeutic regimens/exposure factor or procedure and any comparison(s) described in detail? Were intervening factors described? Q7 = Were outcomes clearly defined and the measurements valid and reliable? Q8 = Was the statistical analysis appropriate for the study design and type of outcome indicators? Q9 = Are conclusions supported by results with biases and limitations taken into consideration? Q10 = Is bias due to study’s funding or sponsorship unlikely? QA = Assessment of scientific soundness of a study; + indicates that most of the scientific soundness criteria questions (including Q2, Q3, Q6, Q7, and at least one additional Yes) have been addressed. Therefore, the study has clearly addressed issues of inclusion/exclusion, bias, generalizability, and data collection and analysis; - indicates that six or more of these scientific soundness criteria questions have not been adequately addressed in the study; Ø indicates that scientific soundness criteria questions, Q2, Q3, Q6, and Q7 were not adequately addressed. Therefore, the study is neither exceptionally strong nor exceptionally weak.

## Supplementary Materials

The following are available online at http://www.mdpi.com/2072-6643/9/3/188/s1.
