# Supplementary material for: Prevalence and Determinants of Overweight and Obesity in Children and Adolescents from Migrant and Seasonal Farmworker Families in the United States—A Systematic Review and Qualitative Assessment
Source: Nutrients. 2017 Feb 24;9(3):188. doi: 10.3390/nu9030188 (PMC5372851; doi:10.3390/nu9030188)
Supplement: Supplementary file 1 [file nutrients-09-00188-s001.docx]

Prevalence and Determinants of Overweight and Obesity in Children and Adolescents from Migrant and Seasonal Farmworker Families in the United States – A Systematic Review and Qualitative Assessment

Yuen Mei Lim, SuJin Song and Won O. Song ^*^

Supplementary A: Search Plan and Results for Primary Research Articles using Template Provided by the Academy of Nutrition and Dietetics’ Evidence Analysis Manual: Steps in the Academy Evidence Analysis Process.

**Search Plan and Results for Primary Research Articles**

**Topic of Interest**: Prevalence and Determinants of Overweight and Obesity (OW/OB) in Children and Adolescents from Migrant and Seasonal Farmworker (MSFW) Families in the United States (US).

**Research Questions**:

1. What is the prevalence of OW/OB in children and adolescents from MSFW families in the US?
2. What are the determinants of OW/OB in children and adolescents from MSFW families in the US?

**Date of Literature Review for the Evidence Analysis**: August 2015

**Inclusion Criteria**:

- Age: Children and adolescents (<20 years)
- Setting: Any
- Health Status: Any
- Nutrition Related Problem/Condition: Children and adolescents with OW/OB status according to the Centers for Disease Control and Prevention 2000 Growth Chart standards.
- Study Design Preference: Any, quantitative over qualitative
- Sample Size: Any
- Sample Type: Children and adolescents (<20 years) from MSFW families
- Study Drop Out Rate: Any
- Publication Year Range: Any up to August 2015
- Authorship: Not applicable since no review articles on this topic exists as of yet
- Language: Articles published in English
- Country: USA
- Content Type: Journal Article, Dissertation
- Discipline: Any

**Exclusion Criteria**:

- Age: Adults (≥20 years)
- Setting: None
- Health Status: None
- Nutrition Related Problem/Condition: Children and adolescents with no OW/OB status
- Study Design Preference: None
- Sample Size: None
- Sample Type: Children and adolescents (<20 years) not from MSFW families
- Study Drop Out Rate: None
- Publication Year Range: After August 2015
- Authorship: Not applicable since no systematic review articles on this topic of interest exists yet
- Language: Articles not published in English
- Country: Any country other than USA
- Content Type: Book/eBook, Book Chapter, Book Review, Government Document, Newsletter, Newspaper Article
- Discipline: None

**Search Terms**: Search Vocabulary

Health Condition: OW, OB

**I**nterventi**on**: Not applicable

Type of Study Design: Any

***Electronic Databases or Search Engine/Method***

**Search engine/method**: Michigan State University Libraries’ Search Plus

Due to the limited research and literature on MSFW population, a search engine/method or electronic database that can perform a quick, comprehensive search for literature from interdisciplinary sources and from multiple library resources while having the ability to apply specific filters based on pre-specified inclusion and exclusion criteria is needed. Among the many electronic databases that exist to perform literature search such as PubMed and Google Scholar, the search engine/method that was selected to perform the systematic review of articles for this research question is Michigan State University Libraries’ Search Plus. Google Scholar was not used because certain filters to exclude certain content types were unavailable. PubMed was also not used because PubMed archives literature specific to the biomedical and life sciences discipline and the number of articles that were retrieved using the specified search terms were too few in numbers.

**Search Terms**: Overweight obesity migrant and seasonal farmworker children

**Filters Applied**:

- Add Results Outside MSU Libraries’ Collections
- Full Text Online

**Hits**: 56

**Articles to Review**: 3

**Total Articles Identified to Review from Electronic Databases or Search Engine/Method**: 56

**Inclusion List**:

**List of Articles Included from Electronic Databases or Search Engine/Method**: 3

1. Kilanowski, J.F.; Gordon, N.H. Making a difference in migrant summer school: testing a healthy weight intervention. *Public Health Nurs* **2015**, *32*, 421-249, 10.1111/phn.12175.
2. Lee, K.; Won, S. Effect of enrollment length in migrant head start on children's weight outcomes. *Health & Social Work* **2015**.
3. Nichols, M.; Stein, A.D.; Wold, J.L. Health status of children of migrant farm workers: farm worker family health program, Moultrie, Georgia. *Am J Pub Health* **2014**, *104*, 365-370.
4. Markowitz, D.L.; Cosminsky, S. Overweight and stunting in migrant Hispanic children in the USA. *Econ Hum Biol* **2005**, *3*, 215-240.

**List of Articles Included from Handsearch or Other Means**: 7

The following articles were retrieved by screening the reference lists of the articles included from the electronic databases or search engine/method and other related articles used in providing background information on the topic of interest and research question.

1. Borre, K.; Ertle, L.; Graff, M. Working to eat: vulnerability, food insecurity, and obesity among migrant and seasonal farmworker families. *Am J Ind Med* **2010**, *53*, 443-462.
2. Grzywacz, J.G.; Suerken, C.K.; Zapata Roblyer, M.I.; Trejo, G.; Arcury, T.A.; Ip, E.H.; Lang, W.; Quandt, S.A. Physical activity of preschool-aged Latino children in farmworker families. *Am J Health Behav* **2014**, *38*, 717-725.
3. Kilanowski, J.F. Lessons learned from a pilot study on the health status of children from itinerant populations. *J Pediatr Health Care* **2006**, *20*, 253-260.
4. Kilanowski, J.F. Patterns and correlates of nutrition among migrant farm-worker children. *West J Nurs Res* **2012**, *34*, 396-416.
5. Kilanowski, J.F.; Ryan-Wenger, N.A. Health status in an invisible population: carnival and migrant worker children. *West J Nurs Res* **2007**, *29*, 100-120.
6. Rosado, J.I.; Johnson, S.B.; McGinnity, K.A.; Cuevas, J.P. Obesity among Latino children within a migrant farmworker community. *Am J Prev Med* **2013**, *44*, S274-S281.
7. Song, W.; Song, S.; Gonzalez, A.; Shin, D.; Nieves, V. Parents' BMI and weight perception are associated with overweight and obesity among children in Michigan migrant and seasonal farm worker families. *Jacobs J Community Med* **2015**, *1*, 1-8.

**List of Excluded Articles with Reason**: 53

| **Excluded Articles** | **Reason for Exclusion** |
| --- | --- |
| Gonzalez, A.; Billings, L.; Shin, D.; Rosenbaum, R.; Song, W. Health disparities in migrant and seasonal farmworker children in Michigan. *J Acad Nutr Diet* **2013**, *113*, A_74. | No full text; only abstract |
| Quandt, S.A.; Grzywacz, J.G.; Trejo, G.; Arcury, T.A. Nutritional strategies of Latino farmworker families with preschool children: identifying leverage points for obesity prevention. *Soc Sci Med* 2014, *123*, 72-81. | Title |
| Weigel, M.M.; Armijos, R.X.; Hall, Y.P.; Ramirez, Y.; Orozco, R. The household food insecurity and health outcomes of U.S.–Mexico border migrant and seasonal farmworkers. *J Immigr Minor Health* 2007, *9*, 157-169. | Title |
| Ward, R.; Chaney, E.H.B.; Rager, R.C.; Burke, S.C. Development of an instrument to assess stress, depression, and coping among Latino migrant and seasonal farmworkers. *Am J Health Stud* 2011, *26*, 236. | Title |
| Grauel, K.; Chambers, K.J. Food deserts and migrant farmworkers: Assessing food access in Oregon's Willamette Valley. *J Ethnobiol* 2014, *34*, 228-250. | Title |
| Clingerman, E.M.; Brown, A. Stress in migrant farmworkers during premigration. *Biol Res Nurs* 2012, *14*, 27-37. | Title |
| Hill, B.G.; Moloney, A.G.; Mize, T.; Himelick, T.; Guest, J.L. Prevalence and predictors of food insecurity in migrant farmworkers in Georgia. *Am J Pub Health* 2011, *101*, 831-833. | Title |
| Brieger, K.M. A dietician's perspective on diabetes among migrant farmworkers. *J Health Care Poor Underserved* 2006, *17*, 469-476. | Title |
| Saenz, C.D. Health risks and health-seeking behaviors of migrant and seasonal farmworkers on the US-Mexico border. ProQuest Dissertations Publishing, 2010. | Title |
| Grzywacz, J.G.; Arcury, T.A.; Trejo, G.; Quandt, S.A. Latino mothers in farmworker families' beliefs about preschool children's physical activity and play. *J Immigr Minor Health* 2016, *18*, 234. | Title |
| Grzywacz, J.G.; Arcury, T.A.; Trejo, G.; Quandt, S.A. Latino mothers in farmworker families' beliefs about preschool children's physical activity and play. *J Immigr Minor Health* 2016, *18*, 234. | Duplicate |
| Johnson, M.H. Contextualizing obesity among Latino farmworkers: a critical analysis of structural and cultural processes affecting farmworker health and nutrition in Central Florida. ProQuest Dissertations Publishing, 2010. | Title |
| Albarran, C.R. Expanding perspectives and gaining leverage: How migrant farmworker women navigate hiv risk in their close, long-term relationships. ProQuest Dissertations Publishing, 2015. | Title |
| Researchers from Oklahoma State University discuss findings in farming (nutritional strategies of Latino farmworker families with preschool children: identifying leverage points for obesity prevention). *Agriculture Week* 2015, 4755. | Title |
| Carvajal, S.C.; Kibor, C.; McClelland, D.J.; Ingram, M.; de Zapien, J.G.; Torres, E.; Redondo, F.; Rodriguez, K.; Rubio-Goldsmith, R.; Meister, J.*, et al.* Stress and sociocultural factors related to health status among US-Mexico border farmworkers. *J Immigr Minor Health* 2014, *16*, 1176-1182. | Title |
| Kilanowski, J.F.; Gordon, N.H. Making a difference in migrant summer school: testing a healthy weight intervention. *Public Health Nurs* 2015, *32*, 421-429. | Relevant but retrieved after Date of Literature Review for the Evidence Analysis |
| Scott, F.J. Examining the impact of a workplace based intervention to reduce obesity and diabetes among immigrant Latino farmworkers. ProQuest Dissertations Publishing, 2014. | Title |
| Lam, M.; Krenz, J.; Palmández, P.; Negrete, M.; Perla, M.; Murphy-Robinson, H.; Spector, J.T. Identification of barriers to the prevention and treatment of heat-related illness in Latino farmworkers using activity-oriented, participatory rural appraisal focus group methods. *BMC Public Health* 2013, *13*, 1004-1004. | Title |
| Renzaho, A.M.N.; Bilal, P.; Marks, G.C. Obesity, type 2 diabetes and high blood pressure amongst recently arrived Sudanese refugees in Queensland, Australia. *J Immigr Minor Health* 2014, *16*, 86-94. | Title; non-USA study |
| Hernández-Valero, M.A.; Bustamante-Montes, L.P.; Hernández, M.; Halley-Castillo, E.; Wilkinson, A.V.; Bondy, M.L.; Olvera, N. Higher risk for obesity among Mexican–American and Mexican immigrant children and adolescents than among peers in Mexico. *J Immigr Minor Health* 2012, *14*, 517-522. | Title |
| Demment, M.M. Understanding the underlying social, maternal, and environmental risk factors for the development of overweight and obesity from birth to adolescence. ProQuest Dissertations Publishing, 2012. | Title; general population |
| Ross, L. Impact of social support on mediating life stressors on abdominal obesity among migrant farm workers. ProQuest Dissertations Publishing, 2014. | Title; adults |
| Queral, C.B. The impact of a nutrition education program on nutrition knowledge and attitudes, as well as food selection, in a cohort of migrant and seasonal farm worker children. ProQuest Dissertations Publishing, 2007. | Title |
| Isanaka, S.; Mora-Plazas, M.; Lopez-Arana, S.; Baylin, A.; Villamor, E. Food insecurity is highly prevalent and predicts underweight but not overweight in adults and school children from bogotá, colombia1-3. *J Nutr* 2007, *137*, 2747. | Title; non-USA study |
| Clingerman, E. Social justice: A framework for culturally competent care. *J Transcult Nurs* 2011, *22*, 334-341. | Title |
| Caballero Deza, A. The health and nutrition of migrant farm workers in South Carolina. ProQuest Dissertations Publishing, 2006. | Title |
| Martin-Fernandez, J.; Caillavet, F.; Lhuissier, A.; Chauvin, P. Food insecurity, a determinant of obesity? - an analysis from a population-based survey in the Paris metropolitan area, 2010. *Obes Facts* 2014, *7*, 120. | Abstract; adults; not MSFW |
| Ortega, M.-I.; Rosales, C.; de Zapien, J.G.; Aranda, P.; Castañeda, A.; Saucedo, S.; Montaño, C.; Contreras, A. Migration, agribusiness and nutritional status of children under five in northwest Mexico. *Int J Environ Res Public Health* 2012, *9*, 33-43. | Title; non-USA study |
| Leung-Heras, J.M. Stories from the front: health care access in the U.S. and Mexico in Mexican migrant farm workers. ProQuest Dissertations Publishing, 2010. | Title; adults |
| Acheampong, I.; Haldeman, L. Are nutrition knowledge, attitudes, and beliefs associated with obesity among low-income Hispanic and African American women caretakers? *J Obes* 2013, *2013*, 1-8. | Title; adults; not MSFW |
| Stott, S.M. A qualitative review of Hispanic food choice: exploring factors for healthy and unhealthy eating among Hispanic children and adolescents living in the U.S. ProQuest Dissertations Publishing, 2015. | Title |
| Heller, M.C.; Keoleian, G.A. Assessing the sustainability of the US food system: a life cycle perspective. *Agr Syst* 2003, *76*, 1007-1041. | Title |
| Lacar, E.S.; Soto, X.; Riley, W.J. Adolescent obesity in a low-income Mexican American district in south Texas. *Arch Pediatr Adolesc Med* 2000, *154*, 837-840. | Title |
| Ramsey, R.; Giskes, K.; Turrell, G.; Gallegos, D. Food insecurity among adults residing in disadvantaged urban areas: potential health and dietary consequences. *Pub Health Nutr* 2012, *15*, 227-237. | Title |
| Sharkey, J.R.; Dean, W.R.; Johnson, C.M. Association of household and community characteristics with adult and child food insecurity among Mexican-origin households in Colonias along the Texas-Mexico border. *Int J Equity Health* 2011, *10*, 19-19. | Title |
| Taylor, T.L. Development and impact of a stage of change bilingual nutrition education program for Hispanics. ProQuest Dissertations Publishing, 1997. | Title |
| Clark, M.L.; Reynolds, S.J.; Hendrikson, E.; Peel, J.L. Asthma prevalence and risk factor assessment of an underserved and primarily Latino child population in Colorado. *J Environ Health* 2014, *76*, 8. | Title |
| Chilton, M.; Black, M.M.; Berkowitz, C.; Casey, P.H.; Cook, J.; Cutts, D.; Jacobs, R.R.; Heeren, T.; de Cuba, S.E.; Coleman, S.*, et al.* Food insecurity and risk of poor health among US-born children of immigrants. *Am J Public Health* 2009, *99*, 556-562. | Title |
| Haldeman, L.A.; Gruber, K.J.; Ingram, K.P. Determinants of food security and diet among rural and urban Latino/Hispanic immigrants. *J Hunger Environ Nutr* 2008, *2*, 67-83. | Title |
| Balagopal, P.; Kamalamma, N.; Patel, T.G.; Misra, R. A community-based participatory diabetes prevention and management intervention in rural India using community health workers. *Diabetes Educ* 2012, *38*, 822-834. | Title |
| Omidvar, N.; Ghazi-Tabatabie, M.; Sadeghi, R.; Mohammadi, F.; Abbasi-Shavazi, M.J. Food insecurity and its sociodemographic correlates among Afghan immigrants in Iran. *J Health Popul Nutr* 2013, *31*, 356. | Title |
| Colby, S.E. The development of peer-led youth theater as a nutrition education tool to promote the healthy traditional Latino diet. ProQuest Dissertations Publishing, 2005. | Title |
| Rush, T.J.; Ng, V.; Irwin, J.D.; Stitt, L.W.; He, M. Food insecurity and dietary intake of immigrant food bank users. *Can J Diet Pract Res: a publication of Dietitians of Canada = Revue canadienne de la pratique et de la recherche en diététique : une publication des Diététistes du Canada* 2007, *68*, 73-78. | Title |
| Ghattas, H.; Sassine, A.J.; Seyfert, K.; Nord, M.; Sahyoun, N.R. Food insecurity among Iraqi refugees living in Lebanon, 10 years after the invasion of Iraq: Data from a household survey. *The Br J Nutr* 2014, *112*, 70-79. | Title |
| Taillie, L.S.; Jaacks, L.M. Toward a just, nutritious, and sustainable food system: the false dichotomy of localism versus supercenterism 1,2. *J Nutr* 2015, *145*, 1380. | Title |
| Kandula, N.R.; Kersey, M.; Lurie, N. Assuring the health of immigrants: What the leading health indicators tell us. *Annu Rev Publ Health* 2004, *25*, 357-376. | Title |
| Currie, D. National public health week celebrated nationwide. *The Nation's Health* 2012, *42*, 18. | Title |
| Interlenghi, G.d.S.; Salles-Costa, R. Inverse association between social support and household food insecurity in a metropolitan area of Rio de Janeiro, Brazil. *Public Health Nutr* 2015, *18*, 2925-2933. | Title |
| Tyler, S.M. "Wake up the knowledge that you have": an assessment of community food security in Fellsmere, Florida. ProQuest Dissertations Publishing, 2014. | Title |
| Jezewski, M.A.; Poss, J. Mexican Americans' explanatory model of type 2 diabetes. *Western J Nurs Res* 2002, *24*, 840-858. | Title |
| Jezewski, M.A.; Poss, J. Mexican Americans' explanatory model of type 2 diabetes. *Western J Nurs Res* 2002, *24*, 840-858. | Duplicate (Article is a commentary) |
| Venci, B.J. Functional limitation and chronic diseases are associated with food insecurity among U.S. Adults: national health interview survey, 2011. ProQuest Dissertations Publishing, 2013. | Title |
| Currie, D. National public health week celebrated nationwide: health advocates nationwide submitted details of their events to the nation's health in April, an alphabetical summary of which follows. *The Nation's Health* 2012, *42*, 18. | Title |

**Number of Primary Articles Identified**: 10

**Number of Review Articles Identified**: 0

**Total Number of Articles Identified**: 10

**Number of Articles Reviewed but Excluded**: 0
